# Pain sensitization as a potentially modifiable factor in people with knee osteoarthritis: insights from behavioral graded activity

**DOI:** 10.3389/fpain.2026.1865792

**Published:** 2026-06-22

**Authors:** Takafumi Hattori, Takako Matsubara

**Affiliations:** Department of Physical Therapy, Faculty of Rehabilitation, Kobe Gakuin University, Kobe, Japan

**Keywords:** behavioral graded activity, knee osteoarthritis, pain sensitization, physical activity, pressure pain threshold, temporal summation of pain

## Abstract

**Introduction:**

Exercise is a first-line treatment for knee osteoarthritis (KOA); however, a substantial proportion of patients show a limited response to standardized exercise programs. Pain sensitization is increasingly recognized as a contributor to poor outcomes. This study examined changes in pain, disability, and pain sensitization after a behavioral graded activity (BGA) program in people with KOA who had not responded to guideline-concordant exercise.

**Methods:**

In this single-arm trial, the pain and activities of daily living subscales of the Knee injury and Osteoarthritis Outcome Score (KOOS) were assessed after a 12-week BGA program. Secondary outcomes included pressure pain threshold (PPT), temporal summation of pain (TSP), and step count. Baseline radiography and magnetic resonance imaging (MRI) were used to characterize structural abnormalities as potential effect modifiers.

**Results:**

Twenty-six participants completed the intervention. Linear mixed models showed significant improvements in KOOS pain and activities of daily living, exceeding the minimal important change. PPT increased at both the knee and forearm, TSP decreased, and physical activity increased. Baseline step count was positively correlated with improvement in KOOS activities of daily living, whereas structural abnormalities and pain sensitization measures were not associated with changes in KOOS outcomes.

**Discussion:**

Clinically meaningful improvements in KOOS were accompanied by concurrent changes in PPT and TSP over the 12-week BGA program in people with KOA who had not responded to guideline-concordant exercise. Future randomized controlled trials are needed to determine whether these sensory changes are attributable to BGA and to identify patient characteristics associated with treatment response.

## 1 Introduction

Knee osteoarthritis (KOA) is a leading cause of pain-related disability and socioeconomic burden worldwide, particularly in aging populations (1). While traditionally regarded as a nociceptive pain condition arising from structural degeneration of the knee joint, growing evidence indicates that structural abnormalities do not consistently correlate with symptom severity (2). This clinical-structural discordance has prompted increasing interest in pain sensitization as a key mechanism underlying chronic pain in KOA (3).

Pain sensitization refers to altered nervous system responses to nociceptive input and is broadly categorized into peripheral sensitization, involving increased responsiveness of primary afferent nociceptors, and central sensitization, reflecting altered central pain processing (4, 5). Quantitative sensory testing (QST), including pressure pain threshold (PPT) and temporal summation of pain (TSP), provides objective indices of pain sensitization; lower PPT and facilitated TSP have been reported in people with KOA (6, 7). In people with KOA, pain sensitization has been implicated in persistent pain and reduced responsiveness to treatment (6, 8, 9).

Exercise therapy is strongly recommended as a first-line intervention for KOA (10) and has been shown to reduce pain through peripheral and central mechanisms, including anti-inflammatory cytokine release, inhibition of nociceptive signal transmission in peripheral nerves, and activation of descending inhibitory systems (11–14). However, despite guideline-concordant exercise, approximately 30% of people with KOA fail to achieve clinically meaningful pain relief (9). Pain sensitization has been proposed as a contributor to this heterogeneity, potentially through heightened susceptibility to exercise-induced hyperalgesia, poorer adherence, and diminished treatment effects (8, 9).

For people exhibiting these sensitization profiles, low-intensity, individually tailored interventions based on gradual exposure to daily activities may offer an alternative strategy (15, 16). Behavioral graded activity (BGA), which emphasizes operant-based, goal-directed physical activity progression rather than prescriptive exercise routines, has shown promise in enhancing physical activity and functional outcomes among chronic pain populations (15, 17). However, the mechanistic impact of BGA on symptoms and pain sensitization in KOA remains poorly understood.

To address this gap, this study examined changes in symptoms and pain sensitization after a 12-week BGA program in people with KOA who had not responded to guideline-concordant exercise. We also examined whether baseline characteristics were associated with treatment response as potential effect modifiers. By focusing on pain processing mechanisms in addition to clinical outcomes, this study aimed to examine whether QST indices of pain sensitization changed during the BGA program.

## 2 Materials and methods

### 2.1 Study design and ethics approval

**Figure 1:**
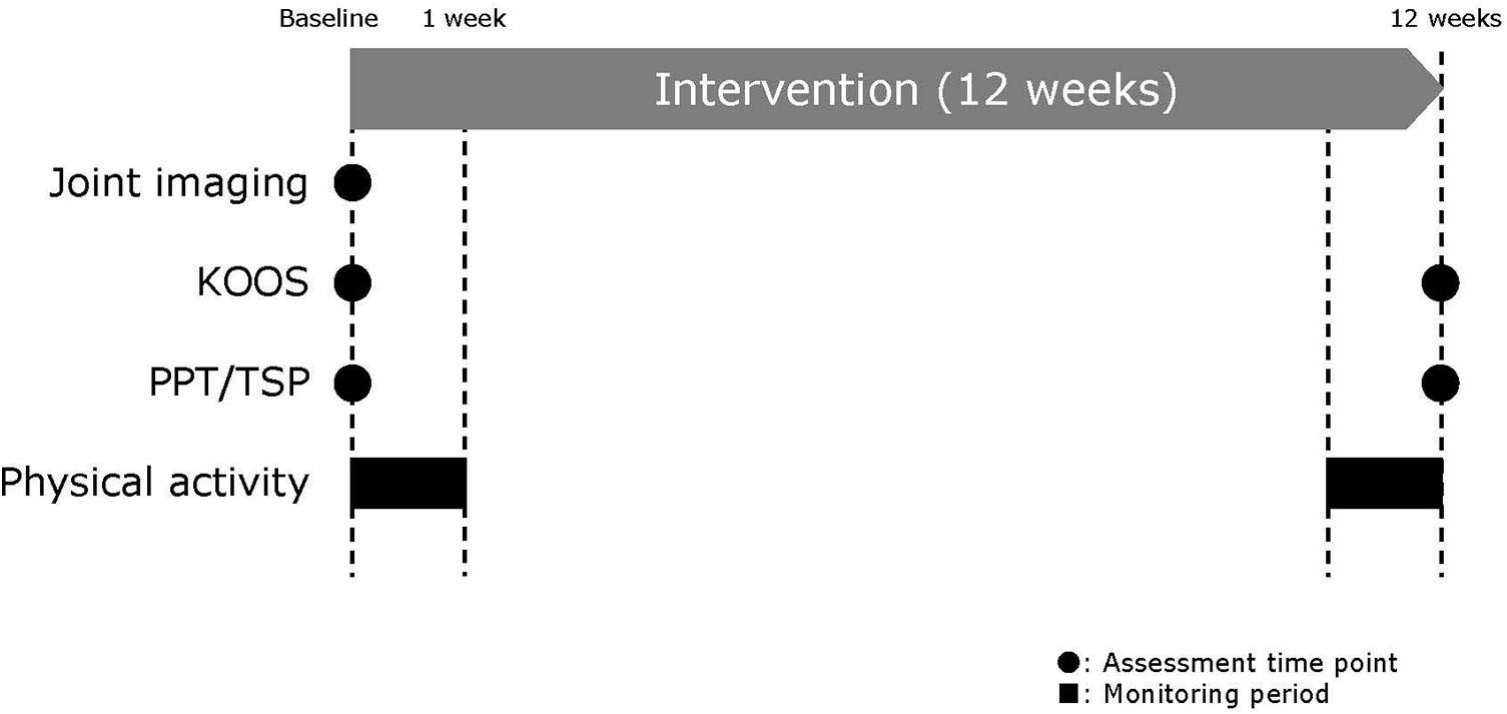
Study protocol KOOS, knee injury and osteoarthritis outcome score; PPT, pressure pain threshold; TSP, temporal summation of pain.

This was a single-arm trial without a control group. The study protocol is shown in Figure 1. To minimize assessor bias, QST was performed by H.T., who was not involved in delivering the intervention. The intervention was delivered by a different physiotherapist.

Ethical approval was obtained from the Institutional Ethics Committee of Kobe Gakuin University in Kobe, Japan (No.: 19–23). Written informed consent was obtained from all participants prior to enrollment. The trial was registered with the University Hospital Medical Information Network Clinical Trials Registry (UMIN000039723).

### 2.2 Participants

Patients diagnosed with KOA who were referred for physiotherapy by an orthopedic surgeon were recruited from Maehara Orthopedic Rehabilitation Clinic in Aichi, Japan. The inclusion criteria were as follows: female sex; age ≥ 50 years; radiographically confirmed KOA [Kellgren–Lawrence (KL) grade ≥ 2]; persistent knee pain for ≥ 6 months; and non-response to a 3-month guideline-concordant exercise program (10). Participants had previously taken part in a prior cohort study (9), in which they completed a 3-month guideline-based exercise program comprising aerobic, resistance, and neuromuscular exercises (three sessions per week, 40 min/session) at the same clinic. Although the prior exercise program was guideline-based, it did not include standardized activity progression procedures grounded in behavior-change principles, as implemented in the present BGA program. Non-response to the prior exercise program was defined as less than a 30% improvement in peak knee pain over the past week, assessed using a visual analogue scale (VAS), according to the Initiative on Methods, Measurement, and Pain Assessment in Clinical Trials (IMMPACT) recommendations (18). In addition, given known sex differences in pain sensitization and the higher prevalence of KOA among women, the study was restricted to female participants (19, 20).

The exclusion criteria included systemic inflammatory diseases (e.g., rheumatoid arthritis); cognitive impairment; severe medical comorbidities (e.g., neurological disorders, neuropsychiatric disorders such as depression or autism, cardiovascular diseases, and cancer); serious hip or ankle pathology (e.g., osteoarthritis and unhealed fractures); leg pain referred from the lumbar spine; previous joint replacement; and use of centrally acting medications (e.g., antidepressants and anxiolytics). In cases of bilateral KOA, the more painful knee was defined as the affected side. Participants were instructed to avoid taking nonsteroidal anti-inflammatory drugs (NSAIDs) on the day of QST.

### 2.3 Baseline assessment

#### 2.3.1 Demographic characteristics

Demographic data, including age, body mass index (BMI), and pain duration, were collected at baseline.

#### 2.3.2 Structural abnormalities

Radiographic severity was graded using the KL classification, which ranges from grade 1 (doubtful OA) to grade 4 (severe OA).

MRI scans were performed using a 1.5-T system (Echelon RX, Nikko Medical, Chiba, Japan) to semi-quantitatively evaluate synovitis and bone marrow lesions (BMLs) using the MRI Osteoarthritis Knee Score (MOAKS) (21). Imaging sequences included T2-weighted fast spin-echo images with fat suppression in the coronal plane (repetition time: 3,544 ms; echo time: 36 ms; field of view: 43.8 × 20 cm; matrix: 256 × 256; slice thickness: 3 mm; slice spacing: 3.3 mm), sagittal plane (repetition time: 5,223 ms; echo time: 80 ms; field of view: 42.2 × 20 cm; matrix: 256 × 228; slice thickness: 3 mm; slice spacing: 3.3 mm), and axial plane (repetition time: 5,868 ms; echo time: 99 ms; field of view: 71.5 × 83 cm; matrix: 320 × 320; slice thickness: 8.5 mm; slice spacing: 9.5 mm). All MRI images were independently interpreted by an orthopedic surgeon with over 20 years of experience in KOA care and MRI evaluation.

Hoffa-synovitis was graded from 0 to 3 (0 = normal, 1 = mild, 2 = moderate, and 3 = severe) according to the hyperintensity on the sagittal fat-suppressed sequence. Hoffa-synovitis was considered to reflect true synovitis (22).

BML size scores were assessed according to the percentage of the volume of the subregion, including any associated cysts, on a 0–3 scale (0 = none, 1 < 33%, 2 = 33%–66%, and 3 > 66%). The BMLs were divided and scored individually in 15 anatomical locations. BMLs were integrated into five regions, including four subchondral regions (medial and lateral femorotibial, medial and lateral patellofemoral) and the subspinous region.

### 2.4 Longitudinal primary outcomes

#### 2.4.1 Knee injury and Osteoarthritis Outcome Score

Pain and disability were evaluated using the pain (9 items) and activities of daily living (17 items) subscales of the Knee injury and Osteoarthritis Outcome Score (KOOS) (23). KOOS is a validated, reliable, and widely used patient-reported outcome measure for people with KOA (23). The minimal important change (MIC) was defined as 12.4 points for the pain subscale and 8.4 points for the activities of daily living subscale (24).

### 2.5 Longitudinal secondary outcomes

#### 2.5.1 Pain sensitization

Pain sensitization was assessed using PPT and TSP. Both were measured with a handheld pressure algometer (Algometer Type II, Somedic AB, Sweden; 1-cm^2^ probe; application rate: 30 kPa/s) (3, 25).

PPT was measured at four standardized locations surrounding the patella of the affected knee: 2 cm distal to the inferomedial and inferolateral patellar edges, and 3 cm medial and lateral to the midpoints of the medial and lateral patellar borders, respectively. The lowest PPT value among these four sites was used to represent localized mechanical hypersensitivity. At the post-intervention assessment, PPT at the knee joint was assessed at the same location that exhibited the lowest PPT at baseline. PPT was also measured at a remote site on the extensor carpi radialis longus to evaluate widespread hypersensitivity.

TSP is commonly used as an experimental index of spinal pain facilitation (26). It refers to the progressive increase in perceived pain during a series of identical nociceptive stimuli and is thought to reflect the temporal summation of nociceptive inputs (27). Its validity and reliability have been established in chronic pain populations (28). TSP was measured at the site with the lowest PPT value on the affected knee. Following a single pressure stimulus, participants rated pain intensity using a VAS, with 0 = “no pain” and 100 = “the worst possible pain.” After confirming the absence of residual pain, 10 identical stimuli were applied at 1-second intervals, and the VAS rating was recorded again after the 10th stimulus. TSP was calculated as the difference between the VAS scores for the 10th and 1st stimulus (25). The pressure intensity used for all stimuli was individualized based on each participant's PPT (3). To avoid prolonged after-sensations, TSP was measured only once at the site.

#### 2.5.2 Physical activity

Daily physical activity was objectively monitored using a waist-worn accelerometer (Kenz Lifecorder, Suzuken Co., Ltd., Nagoya, Japan) for 7 consecutive days at baseline and post-intervention (29). The mean daily step count was calculated based on valid wear days.

### 2.6 Intervention

The BGA program consisted of a personalized, low-intensity, and progressively graded daily activity plan tailored to each patient's functional limitations and grounded in operant conditioning principles (15, 30, 31). The intervention was centered on each patient's primary complaint and personal life goals (17). To ensure treatment fidelity, the program was delivered by physiotherapists trained in both the theoretical framework and practical delivery of BGA.

**Figure 2:**
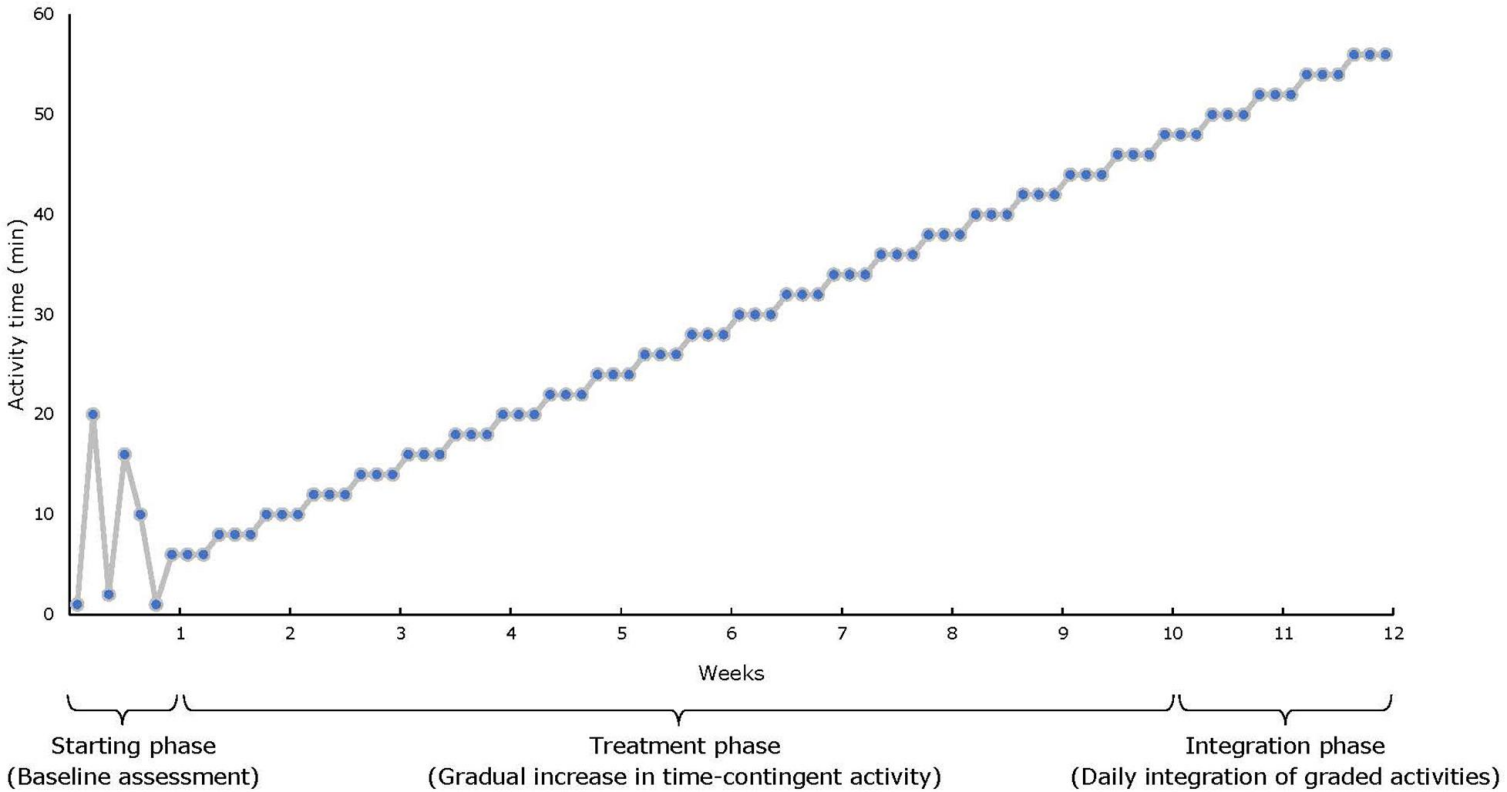
Illustrative model of time-contingent progression of target activity during the behavioral graded activity program.

The program comprised three phases: a starting phase, a treatment phase, and an integration phase (Figure 2). In addition, all participants received pain education throughout the program as an integrated component of the intervention, covering: (1) the distinction between nociception and pain and its clinical relevance; (2) adaptive and dynamic properties of nociceptors and other elements of the pain system; and (3) biopsychosocial mechanisms contributing to the experience and persistence of chronic pain and implications for treatment strategies (32).

#### 2.6.1 Starting phase

During the first week, baseline activity levels were assessed. Physiotherapists evaluated task-specific activity tolerance and fear of movement. Patients’ pain-related beliefs were explored using the vicious circle model (33). Physiotherapists and patients collaboratively established long- and short-term activity goals and selected target tasks amenable to graded progression. Typical target tasks included leisure/hobby activities (e.g., gardening, recreational outings), household activities (e.g., cooking, shopping), and mobility tasks (e.g., walking, stair climbing). Individualized time-contingent quotas were set based on task-specific tolerance.

#### 2.6.2 Treatment phase

Over 12 weeks, the program was delivered once weekly in the outpatient setting (approximately 40 min/session). Activities were progressed using a time-contingent approach, with quotas increased systematically toward preset goals. Participants were instructed to adhere to the prescribed quotas, without overperforming or underperforming them. Progression was paused only when signs suggestive of an active inflammatory process were suspected, after which progression resumed at a lower level. Parameters such as frequency, intensity, repetitions, and duration were adjusted according to individual capacity. Participants recorded daily performance in an activity diary to facilitate self-monitoring and encourage adherence. Successful task engagement was positively reinforced, with regular modifications based on functional performance and patient feedback.

When walking was selected as the target activity for a long-term goal such as traveling, the initial quota was set slightly below the participant's baseline tolerance and then increased gradually. For example, when the preset goal was 30 min of continuous walking, the walking quota was increased by approximately 1 min every 3 days so that the participant could reach the target duration by the end of the 12-week program. Similarly, for hobby and household activities, the number of repetitions or task duration was gradually increased according to predetermined quotas.

#### 2.6.3 Integration phase

In the later weeks of the intervention, participants were encouraged to integrate the progressed activities into daily life to support longer-term adherence and maintenance of functional gains.

### 2.7 Data analysis

Statistical analyses were performed using JMP (SAS Institute Inc., Cary, USA). Normality was assessed using the Shapiro–Wilk test. As the continuous variables were not normally distributed, baseline characteristics were reported as median (IQR) for continuous variables and *n* (%) for categorical variables.

Longitudinal analyses were performed using linear mixed models to examine changes in KOOS scores, QST, and physical activity following the 12-week BGA program. Time (pre vs. post) was modeled as a fixed effect, and a participant-level random intercept was included. Model parameters were estimated using restricted maximum likelihood with unbounded variance components. In this single-arm trial, the primary outcomes were changes from pre to post in the pain and activities of daily living subscales of the KOOS; secondary outcomes included QST measures and step counts. Model assumptions were checked by inspection of residuals.

Sample size was calculated based on the MIC of KOOS (pain: 12.4 points; activities of daily living: 8.4 points) (24) and the standard deviations of the KOOS in a previous study (pain: 8.96; activities of daily living: 10.29; *n* = 25) (34). A Bonferroni-adjusted two-sided *α* of 0.025 was applied for the two primary outcomes, with 90% power. The required number of completers was 10 for the KOOS pain subscale and 19 for the KOOS activities of daily living subscale; therefore, 19 completers were targeted. Assuming 30% attrition, the planned recruitment target was 28 participants. Secondary outcome analyses were exploratory; therefore, *p*-values for secondary endpoints were not adjusted for multiple comparisons.

Potential effect modifiers were examined in an exploratory analysis using Pearson’s or Spearman’s rank correlations between changes in KOOS pain and KOOS activities of daily living and baseline variables, including demographics, KL grade, MOAKS score, QST, and physical activity.

## 3 Results

A total of 32 patients completed the baseline assessment and initiated the intervention. Six patients discontinued outpatient visits for personal reasons; therefore, 26 patients were included in the final analysis. Table 1 presents their baseline characteristics. No adverse events related to the intervention were reported. There were no significant differences between completers and dropouts in age (*p* = 0.52), BMI (*p* = 0.11), or pain duration (*p* = 0.25). Twenty participants (76.9%) used NSAIDs as needed for pain exacerbations before the BGA program and continued to use them throughout the intervention period. NSAIDs use was monitored throughout the intervention period and was reduced according to symptom improvement. After the intervention, NSAIDs use was reported by eight participants (30.8%).

**Table 1:** Baseline characteristics.

Variables	Median (IQR) or *n* (%)
n	26
Age	70.5 (61.8, 75.8)
BMI, kg/m^2^	24.8 (21.7, 26.8)
Pain duration, months	42.0 (24.0, 94.5)
Use of NSAIDs	20 (76.9)
KL grade	2:18 (69.2), 3:8 (30.8), 4:0 (0)
MOAKS Synovitis, score	1.0 (0, 1.0)
MOAKS BML, score	4.0 (1.0, 5.8)
KOOS pain	52.8 (45.1, 65.3)
KOOS activities of daily living	73.5 (62.1, 82.0)
PPT at the knee joint, kPa	226.0 (135.3, 322.3)
PPT at the forearm, kPa	309.5 (212.8, 366.5)
TSP, points	20.5 (10.3, 32.3)
Step count, steps/day	2,530.5 (2,031.5, 3,588.3)

BMI, body mass index; NSAIDs, nonsteroidal anti-inflammatory drugs; KL, kellgren–lawrence grade; MOAKS, MRI osteoarthritis knee score; PPT, pressure pain threshold; TSP, temporal summation of pain.

### 3.1 Changes in longitudinal outcomes

 Table 2 presents the results of the longitudinal outcomes. Significant improvements were observed in the KOOS pain (estimated mean change: 15.44, 95% CI: 7.25 to 23.63) and the KOOS activities of daily living subscale (estimated mean change: 11.78, 95% CI: 2.12 to 21.44). PPT increased significantly at both the affected knee (estimated mean change: 169.26, 95% CI: 101.90 to 236.63) and the forearm (estimated mean change: 66.92, 95% CI: 7.57 to 126.26). TSP also decreased significantly (estimated mean change: −12.4, 95% CI: −19.77 to −5.07). Step count showed a significant increase as well (estimated mean change: 1,628.88, 95% CI: 819.62 to 2,438.14).

**Table 2:** Linear mixed models for within-group change in longitudinal outcomes.

Variables	Estimated mean change	SE	t	p	95% CI
KOOS pain	15.44	4.07	3.79	<0.001	7.25 to 23.63
KOOS activities of daily living	11.78	4.80	2.45	0.01	2.12 to 21.44
PPT at the knee	169.26	33.5	5.05	<0.001	101.90 to 236.63
PPT at the forearm	66.92	29.53	2.27	0.02	7.57 to 126.26
TSP	−12.4	3.65	−3.40	0.001	−19.77 to −5.07
Step count	1,628.88	402.90	4.04	<0.001	819.62 to 2,438.14

KOOS, knee injury and osteoarthritis outcome score; PPT, pressure pain threshold; TSP, temporal summation of pain.

### 3.2 Correlation analysis

Table 3 presents the correlations between baseline measures and changes in KOOS scores. Only the baseline step count was positively correlated with changes in the KOOS activities of daily living subscale, whereas no other variables, including radiographic and MRI findings, were associated with changes in KOOS outcomes. This finding suggests that higher baseline physical activity may be associated with greater functional improvement, whereas baseline structural abnormalities and pain sensitization measures did not explain treatment response in this cohort.

**Table 3:** Correlation between change in KOOS scores and baseline variables.

Variables	Correlation coefficient	p
*Δ*KOOS pain		
Age	−0.03	0.85
BMI	0.03	0.88
Pain duration	0.16	0.42
Use of NSAIDs	0.19	0.34
KL grade	−0.03	0.87
MOAKS Synovitis	0.20	0.30
MOAKS BML	0.29	0.14
PPT at the knee joint	0.19	0.33
PPT at the forearm	0.35	0.07
TSP	−0.14	0.48
Step count	0.27	0.17
*Δ*KOOS activities of daily living		
Age	−0.27	0.17
BMI	0.37	0.06
Pain duration	0.09	0.65
Use of NSAIDs	0.20	0.32
KL grade	0.21	0.30
MOAKS Synovitis	−0.07	0.70
MOAKS BML	0.16	0.42
PPT at the knee joint	0.35	0.07
PPT at the forearm	0.18	0.35
TSP	0.00	0.97
Step count	0.43	0.02

Values are presented as correlation coefficients and *p*-values. Changes in KOOS activities of daily living were significantly correlated with baseline physical activity, whereas no other clinical, structural, or sensory variables showed significant associations.

BMI, body mass index; NSAIDs, nonsteroidal anti-inflammatory drugs; KOOS, knee injury and osteoarthritis outcome score; KL, kellgren–lawrence grade; MOAKS, MRI osteoarthritis knee score; PPT, pressure pain threshold; TSP, temporal summation of pain.

## 4 Discussion

This exploratory study investigated changes in pain, disability, and QST measures following a BGA program in people with KOA who had not responded to guideline-concordant exercise therapy. Rather than evaluating the intervention effect itself, the primary aim was to examine whether symptoms and pain sensitization changed over the intervention period. A secondary aim was to examine whether baseline characteristics were associated with treatment response.

The novelty of this study lies in the observation that QST indices of pain sensitization changed in parallel with the intervention period. These findings suggest that gradually progressive daily activity may be associated with changes in pain sensitivity. Notably these sensory changes were observed in patients who had shown a poor response to conventional exercise therapy, suggesting that sensitization may change over the course of a structured activity-based program in this subgroup. However, because this was a single-arm study without a control group and the QST outcomes were exploratory secondary endpoints that were not adjusted for multiple comparisons, these findings should be interpreted cautiously, and the subsequent discussion is framed as exploratory.

Lower PPTs at the affected and remote sites are considered to reflect localized or widespread hypersensitivity, whereas facilitated TSP reflects increased excitability of spinal dorsal horn neurons (26). In KOA, localized mechanical hyperalgesia has been linked to inflammatory mediators such as C-reactive protein and interleukin-6 (35). Additionally, persistent nociceptive input from the osteoarthritic joint may contribute to spinal and supraspinal sensitization through excitatory neurotransmitters such as glutamate, substance P, and calcitonin gene-related peptide, with glial activation in the dorsal horn and dorsal root ganglia also implicated in this process (36–38). Previous cohort studies have identified pain sensitization as a negative prognostic factor for exercise in KOA, with specific QST measures predicting non-responders (8, 9, 39). The baseline sensory profiles of participants in the present study were consistent with these high-risk phenotypes (9), supporting the interpretation that this sample represented a sensitized, exercise-nonresponsive subgroup. Nevertheless, changes in both PPT and TSP were observed during the intervention period, indicating that QST indices of pain sensitization were not entirely stable in this exercise-nonresponsive subgroup.

These sensory changes may be interpreted in relation to previously proposed pathways linking physical activity and pain modulation. Peripheral mechanisms include reductions in inflammatory signaling and alterations in nociceptor sensitivity (11, 13, 40), whereas central mechanisms involve activation of endogenous pain inhibitory systems, including opioid, endocannabinoid, and serotonergic pathways (13, 41, 42). Importantly, excessive or poorly dosed exercise may exacerbate pain in sensitized individuals (25, 43). In contrast, low-intensity graded exposure, which is characteristic of BGA, may enable engagement of inhibitory systems without triggering symptom flares. In particular, the observed improvements in remote PPT and TSP may be compatible with mechanisms beyond peripheral processes, including central modulation.

Our study found that improvement in KOOS activities of daily living was modestly positively correlated with baseline step count. This finding is consistent with previous evidence suggesting that higher objectively measured physical activity levels may be associated with greater exercise-induced hypoalgesia in older adults (44). In contrast, structural abnormalities, including BMLs, were not associated with changes in KOOS outcomes in a previous study (45), which is consistent with the present findings. Since BGA targets behavioral determinants of functioning, such as activity patterns and self-efficacy, baseline activity may better explain differences in treatment response than radiographic and MRI findings or QST measures. However, these null findings should be interpreted cautiously, as restricting the cohort to non-responders may have reduced variability in baseline structural and sensory characteristics and limited the ability to detect associations.

The clinical relevance of BGA lies in its ability to deliver gradual, individualized exposure to meaningful daily activities while minimizing symptom exacerbation (15, 16). Behavioral graded approaches have been associated with improved exercise adherence, greater activity engagement, and better long-term functional outcomes across chronic pain conditions, including low back pain, complex regional pain syndrome, and osteoarthritis (46–48). In people with KOA who have pain sensitization and do not tolerate conventional exercise, BGA may be a feasible option for resuming activity. However, randomized controlled trials are needed to determine whether it affects pain processing mechanisms. Psychological assessments should also be incorporated, because factors not measured in the present study may influence both activity engagement and pain sensitization. Identifying which psychological factors improve during the intervention may be important for advancing the clinical application of BGA.

Several limitations should be acknowledged. First, because this was a single-arm trial without a control group, we cannot draw causal conclusions about the observed changes in pain sensitization. The pre–post changes may also have been influenced by regression to the mean, normal symptom fluctuation, familiarization with repeated QST, and expectancy or placebo effects related to participation in a structured program. In particular, repeated QST exposure may lead to habituation or time-dependent changes in sensory responses, making it difficult to distinguish true changes in pain sensitivity from measurement-related effects in the absence of a control group. Moreover, the potential influence of NSAIDs use on outcomes cannot be ruled out. Although participants had previously shown insufficient improvement despite NSAIDs use in the preceding cohort study, changes in NSAIDs use during the intervention may still have influenced pain, function, or QST outcomes. Second, because of the small sample size, the exploratory analyses of baseline predictors may have been underpowered and require confirmation in larger, multicenter samples. Future studies should use multivariable models that adjust for relevant covariates potentially influencing treatment response. Third, restricting the cohort to individuals who had not responded to guideline-concordant exercise therapy may have reduced variability in baseline structural and sensory measures (range restriction), potentially attenuating associations between baseline characteristics and changes in KOOS outcomes. Fourth, psychological confounders, such as catastrophizing, kinesiophobia, and self-efficacy, should be incorporated into future multidimensional models. Finally, only female participants were recruited from a single outpatient clinic, which may limit generalizability.

## 5 Conclusion

In patients with KOA who did not respond to guideline-concordant exercise therapy, changes in both symptoms and QST indices were observed during the 12-week BGA program. Because this was a single-arm trial, these findings should be interpreted as hypothesis-generating, and randomized controlled trials are warranted. Further research is also needed to clarify the clinical relevance of BGA and to identify determinants of treatment response, including which patients are most likely to benefit.

## Data Availability

The raw data supporting the conclusions of this article will be made available by the authors, without undue reservation.
